# Single-cell RNA sequencing to track novel perspectives in HSC heterogeneity

**DOI:** 10.1186/s13287-022-02718-1

**Published:** 2022-01-29

**Authors:** Pan Zhang, Xiang Li, Chengwei Pan, Xinmin Zheng, Bohan Hu, Ruiheng Xie, Jialu Hu, Xuequn Shang, Hui Yang

**Affiliations:** 1 School of Life Sciences, Northwestern Polytechnical University, Xi’an, 710072 Shaanxi China; 2 Research Center of Special Environmental Biomechanics and Medical Engineering, Northwestern Polytechnical University, Xi’an, 710072 Shaanxi China; 3 College of Engineering, Northwest A&F University, Yangling Demonstration Area, Xianyang, 712100 Shaanxi China; 4 College of Engineering, University of Delaware, Newark, DE 19716-5600 USA; 5 School of Computer Science, Northwestern Polytechnical University, Xi’an, 710072 Shaanxi China; 6 Biomedical Engineering and Immunology, Northwestern Polytechnical University, #127 West Youyi Road, Xi’an, 710072 Shaanxi China

**Keywords:** Hematopoietic stem and progenitor cells, Single-cell RNA sequencing, Heterogeneity, Hematopoietic hierarchy, Bone marrow microenvironment, Malignant hematopoiesis

## Abstract

As the importance of cell heterogeneity has begun to be emphasized, single-cell sequencing approaches are rapidly adopted to study cell heterogeneity and cellular evolutionary relationships of various cells, including stem cell populations. The hematopoietic stem and progenitor cell (HSPC) compartment contains HSC hematopoietic stem cells (HSCs) and distinct hematopoietic cells with different abilities to self-renew. These cells perform their own functions to maintain different hematopoietic lineages. Undeniably, single-cell sequencing approaches, including single-cell RNA sequencing (scRNA-seq) technologies, empower more opportunities to study the heterogeneity of normal and pathological HSCs. In this review, we discuss how these scRNA-seq technologies contribute to tracing origin and lineage commitment of HSCs, profiling the bone marrow microenvironment and providing high-resolution dissection of malignant hematopoiesis, leading to exciting new findings in HSC biology.

## Introduction

Because of the self-renewal and multipotency properties of hematopoietic stem cells (HSCs), the adult hematopoietic system is maintained and balanced throughout the lifespan of an individual. During embryonic development, the aorto-gonad-mesonephros (AGM) region has been confirmed as where definitive hematopoiesis first emerges. Thereafter, hematopoiesis shifts to the fetal liver, and subsequently to the bone marrow [1]. Adult bone marrow (BM) niche acts as an important scaffold for a pool of hematopoietic cells, including HSCs and offspring hematopoietic progenitor cells (HPCs), and extrinsically orchestrates their functions [2]. As the functional environment to HSCs, BM niches integrate a set of extrinsic signals derived from cells alongside HSC and biophysical cues from the extracellular matrix (ECM) required for cell maintenance [3]. Distinct BM niches, such as the generally well-defined endosteal and perivascular, have several discrete anatomical localizations within the marrow. They are believed to serve a differing role for HSCs behaviors in an extrinsic manner, including self-renewal, migration, proliferation, and multilineage capacity [4–6]. From the aspect of cellularly heterogeneity, intensive studies have been conducted to clarify the complexity behind these functional niches by classical genetic approaches, such as Cre-mediated lineage tracing and deletion of molecular factors [7, 8]. However, labeling of impure populations by single markers has resulted in the ambiguities concerning cell type identities, localization and cellular sources of cytokines. Label-free approaches in this field have been expected to elucidate the exact location and the contributions of specific niche populations regulating hematopoiesis and its perturbation by diseases.

Successful bone marrow transplantation depends on the state of these hematopoietic stem and progenitor cells (HSPCs), which contribute to long-term reconstitution of the whole blood system and simultaneously maintain hematopoiesis in the short and intermediate terms after transplantation. As previously reported, downstream lineage-restricted hierarchies can be generated directly from HSCs, which originally keep quiescent and are recruited into cell cycle under stress [9]; the latest evidence suggested that the primary appearance of steady-state blood cells may attribute to the successive recruitment of long-lived HPC populations, rather than HSCs themselves [10]. But the complexity of the pathway through which mature hematopoietic lineages are developed still remains controversial currently. Understanding the relative contributions of these hematopoietic stem and progenitor populations, as well as pathogenic mechanisms of hematological disorders, will provide a view for clinical problems.

## The importance of profiling HSCs in light of single cell

Based on the cellular heterogeneity of biological tissues that can be influenced by both physiological and pathological conditions, numerous new insights for understanding cell-to-cell variation and cellular evolutionary relationships have been presented. Genome-wide measurements conduce to characterize the mechanisms that give rise to diverse biological processes and how they go awry in disease [11]. In general, traditional ensemble-based sequencing technologies reveal an average state of gene expression across a large population, and thus lose cellular heterogeneity information. Tiny distinctions between individual cells are still concealed, which could be important to decision-making processes, even within a ‘homogeneous’ cell population [12]. Analyses of gene expression would be performed using single cells. In addition, owing to the rarity of some cell types, large numbers of animals are required for sufficient starting materials, such as thousands or even millions of cells for bulk measurement [13].

**Fig. 1:**
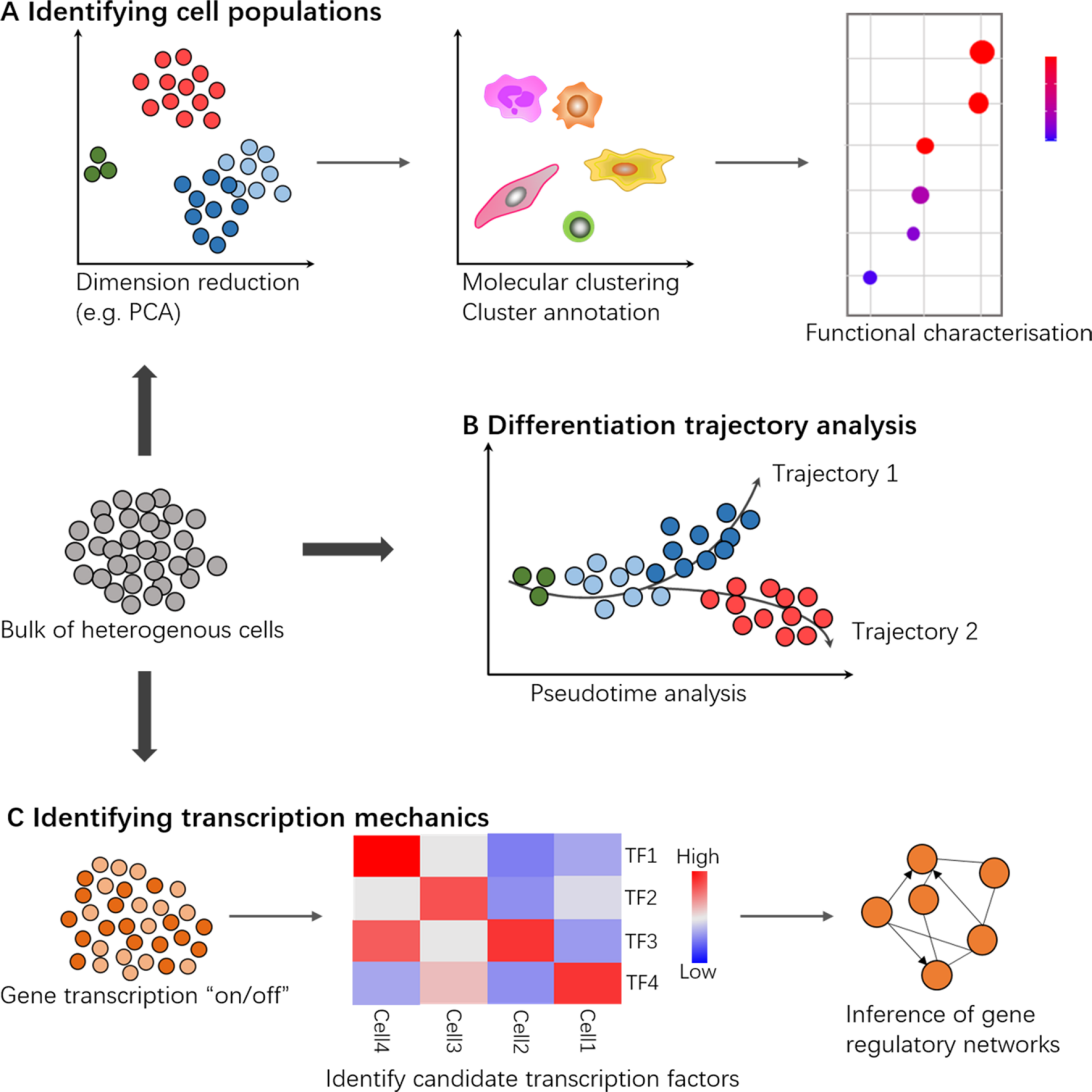
Typical applications of single-cell RNA sequencing. **A** Identifying cell population. scRNA-Seq datasets are processed through dimension reduction techniques to ease visual evaluation. Molecular clustering enables the identification of heterogeneous cell subtypes and novel populations. Clusters can be further annotated by their gene expression characteristics. **B** Differentiation trajectory analysis. Pseudotime analysis basing on scRNA-Seq datasets orders single cell along the “time-series” axis that represents dynamic cell state transitions, such as differentiation or signaling responses to an external stimulus. On the cell developmental trajectories map, special genes that drive branching events can be highlighted. **C** Identifying transcription mechanics. Cell transcription state and candidate transcription factors can be exploited to guide the reconstruction of gene regulatory networks, which suggest critical insights into transcriptional dynamics and the mechanisms driving cellular heterogeneity

HSCs are defined at the single-cell level. But the multipotent progenitor pool is heterogeneous. Accurately assessing cell function both at the population and individual cell levels is essential to the research of HSPCs. Single-cell RNA sequencing (scRNA-seq) technologies, which provide an unbiased view of the transcriptome information at single-base resolution, therefore have the advantages of highlighting cell heterogeneity, revealing cell transcriptional status and shedding light on cell developmental process (Fig. 1). Trajectory inference based on scRNA-seq data can order cells along a putative trajectory according to their distance from a predefined starting point. That can be used to reveal the molecular dynamics of various cell types, delineate cell differentiation paths, and reveal cell fate changes [14]. Practically, these technologies often require far less input material, and researchers always have the flexibility to specify the number of cells to be sequenced. Recent years, as the reduction in sequencing cost, scRNA-seq has been rapidly adopted to comprehensively dissect HSPC functions. In this review, we focus on scRNA-seq applications in the field of HSPC research and discuss the recent progress as well as future perspectives of such techniques in HSPC research.

## Groundbreaking insights into HSC by single-cell analysis

Single-cell transcriptomics is widely expected to answer the key questions about the nature of HSCs in just the past few years. Using sc-RNA seq, putative differentiation routes and dynamic gene expression profile during hematopoietic origin, lineage decision at various developmental stages, and disease are revealed, and the links between molecular features and cellular function are gained [12]. In addition, sc-RNA seq has already provided an unprecedented opportunity for discovering new cell types [15]. As we summarized below, the increasing new landscape of HSC may challenge our preconceptions about hematopoiesis, or provide a more holistic understanding of blood disorders [16].

### Tracing the origin of HSCs

In mammals, the primitive type of HSCs is detected within the yolk sac (YS) before circulation is initiated. As organogenesis commences, hematopoietic clusters are specifically generated during a conservative developmental process termed endothelial‐to‐hematopoietic transition (EHT) in the developing AGM region, which is conserved across vertebrates, including zebrafish, mice, and humans [17, 18]. Embryonic hematopoiesis occurs in multiple anatomical sites including the placenta, the thymus, and the fetal liver (FL). Adult-type HSCs arise, proliferate, and migrate between these multiple sites, and ultimately colonize into the bone marrow, which provides environmental niches to support functional HSCs [19].

Single-cell sequencing technologies have been adopted to investigate the dynamic network features of hemogenic endothelial cells (HECs), intra-aortic hematopoietic cluster (IAHC) cells and primitive HSC production in vertebrate embryos. Baron et al. identified two types of pre-HSCs (Rac2 clusters and Cdh5 clusters) and committed progenitors during EHT and IAHC formation. Type I and type II pre-HSCs have a very similar transcriptome. In the aorta of E10 and E11 embryos, genes and transcription factors (TFs) involved in IAHC formation are found activated, which silence the endothelial program and initiate the hematopoietic program. Transcriptomes of pre-HSCs and HECs, non-HECs and EHT cells reflect the continuum of differentiation occurring between these successive related groups. Pathways such as interleukins, TGF, chemokines, and/or BMP are more active in the endothelium and IAHC cells, while pathways involving hemostasis and cell surface interactions at the vascular wall are activated in the progenitor population. IAHCs in the ventral and dorsal aorta also have similar transcriptomes at both embryonic day E10 and E11, but most molecular changes occurred in ventral might make more contributions in pre-HSCs maturation [20].

Noteworthy, high-precision single-cell transcriptomics of the relevant endothelial cell (EC) populations showed that the embryonic endothelial evolution toward first HSCs even traces back to E8. In the AGM region, primitive vascular ECs experience two-step, namely an initial arterial fate choice followed by a hemogenic fate conversion, and then become HSC-competent HECs, whereafter, the number of putative HSC-primed HECs peaked at E10 and sharply decrease thereafter [21]. Before HEC formation, a distinct pre-HE was found accumulated between arterial ECs and HECs. The intermediate stage characterized by pre-HE suggests a bottleneck between pre-HE and HEC, and Runx1 promotes cells pass through the bottleneck and transition to HECs. Separated developmental trajectories as HECs differentiate into IAHCs depicts a wave of CD45^+^ lympho-myeloid-biased progenitors, which might colonize the FL and thymus prior to HSCs [22].

Iterative single-cell approaches captured the transcriptome of the first functional HSCs as they are generated from Gata2-expressing IAHC cells. In the mouse embryo, refined CD31, cKit, and CD27 phenotypic parameters can define all HSCs, and the first cells achieving HSC function are stochastically localized to aortic clusters containing 1–2 cells [23]. Wang et al. defined the important role of autophagy-essential genes in HSC emergence. According to the scRNA-seq datasets of 5 population cells related to HSC maturation on mouse embryogenesis, the transcription activity of autophagy-essential genes has a sharp increase when endothelial precursors specify into pre-HSC, and such autophagy in pre-HSCs maybe negatively correlated with the activity of NOCTH signaling [24]. A time course analysis at single-cell resolution, together with upregulated interferon-induced genes and cell-cycle genes, emphasized the effect of interferon exposure during the maturation of FL HSCs from pre-HSCs and indicated cycling FL HSCs are already primed to develop into adult quiescent long-term HSCs (LT-HSCs) [25]. Relying on the single-cell long non-coding RNA (lncRNA) landscape of embryonic HSC ontogeny, H19 was defined as a pivotal promoter for HSC generation during the EHT, through regulating the demethylation of hematopoietic transcription factors in pre-HSCs, including Runx1 and Spi1 [26].

Besides, applications of single-cell sequencing technologies are convenient for tracing the generation of human hematopoiesis. Liu Bing firstly constructed a molecular landscape covering the entire course of the EHT process [27]. The developmental trajectory from arterial ECs via HSC-primed HECs to HSPCs in the AGM region uncovered a distinct expression pattern of genes including GJA5, EMCN, RUNX1T1, and PROCR. They were transiently over-expressed upon the initial step of hemogenic fate choice of arterial ECs, but were downregulated in HSPCs. The molecular features distinguished the HSC-primed HECs from the ones related to transient hematopoiesis. Meanwhile, a distinct HEC population that lacked the arterial feature was segregated from the ECs at CS10 stage. In contrast to late HSC-primed HEC, the emergence of early HEC may be independent of arterial vessels and thus should stand aside the generation of multi-potential HSPC [27, 28]. Therefore, tracing pre-HSC at single-cell level precisely, particularly the scarce and transient precursors thereof, can reveal profound new discoveries related to the origins of HSCs.

### Reconstructing hematopoietic hierarchy by scRNA-seq

#### Hematopoietic hierarchy in fetal liver

Fetal liver has been demonstrated as pivotal hematopoietic site where definitive HSCs first colonize before birth [29]. Significant transcriptional heterogeneity of HSPCs within developmental liver has been elaborated, replenishing the exact hematopoietic snapshots during fetal development [30, 31]. Observations by Ranzoni et al. have signified that even immunophenotypically homogeneous populations (such as HSCs, MPPs, CMPs, GMPs, MEPs, and CLPs) are comprised of more than 10 different transcriptionally defined populations, and thus, investigators should critically interrogate currently used CD markers in the context of fetal hematopoietic progenitors. Detailed scRNA-seq maps allow for refined sorting strategy to enrich for human fetal-derived HSC in vivo and hPSCs-derived hematopoietic progenitors in vitro [30, 32]. Developmental trajectory provides further evidence that human fetal HSCs have multilineage output to MEMPs, granulocytes and LMP, differing from adult blood that are mainly unilineage [33]. The initial lineage-priming program is accelerated by the chromatin accessibility of some specific TFs (such as GATA1) precedes their gene activity. During migrating from fetal liver to bone marrow, highly proliferative HSCs shift to quiescent with downregulation of genes related to actin cytoskeleton remodeling, cell adhesion, and migration [30]. It reflects the propensity of liver HSCs to migrate to other tissue [34], and their lineage-committed fate will be further modulated by the niche into which they home [35, 36].

Comparative analysis of neonatal HSC between human and mouse from developing embryos enables us to comprehend each of the pivotal steps along HSC development across species. Despite the major cell types and gene regulatory networks related to lineage differentiation of key fetal liver cell families (endoderm-derived lineages, erythroid lineages, non-erythroid hematopoietic lineages, and mesoderm-derived non-hematopoietic lineages) are relatively conserved between humans and mice over developmental time, there are differences observed on HSPC-derived lineage composition, including a large percentage of neutrophils in mouse fetal liver but not in human [37, 38]. Several novel perspectives have also been acquired recently by an integrative transcriptomic analysis of HSCs from AGM region, fetal liver and adult BM [38]. Human AGM-HSPC populations featured by arterial marker GJA5 and mouse pre-HSC populations display similar endothelial- or hematopoietic-biased characteristics, indicating the arterial endothelial cell derivation of HSCs in both human [27] and mouse AGM [21, 22]. The other major AGM-derived populations in human with mature HSC nature are clustered together with liver samples. In contrast, mouse HSC-competent populations in AGM and those in fetal liver display apparent molecular differences and are clearly separated, suggestive of the maturation of HSC occurs early before colonizing the fetal liver in human. Whereas it was not the case in mouse, as AGM region, fetal liver and adult BM HSCs from mouse individually form three distinct parts on the single-cell visualized atlas [38]. Consistent with above report, another dataset has provided that HSCs from E16.5 liver and P7 bone marrow are individually clustered, but P0 bone marrow and liver HSC populations are projected onto single cluster that is distinct from the E16.5 and P7 HSCs. The two samples from P0 mouse embryos share adult identity scores that closely resembled adult HSCs, and individual neonatal HSCs/HPCs co-express fetal and adult transcripts [39]. Such fetal-to-adult transition occurs progressively along a continuum timeline in which adult-specific enhancers are gradually activated, both in mouse and human [40]. These findings prove the notion for the gradual maturity of adult-like HSPCs that begins before birth.

#### New interpretation of adult hematopoietic lineage tree

**Fig. 2:**
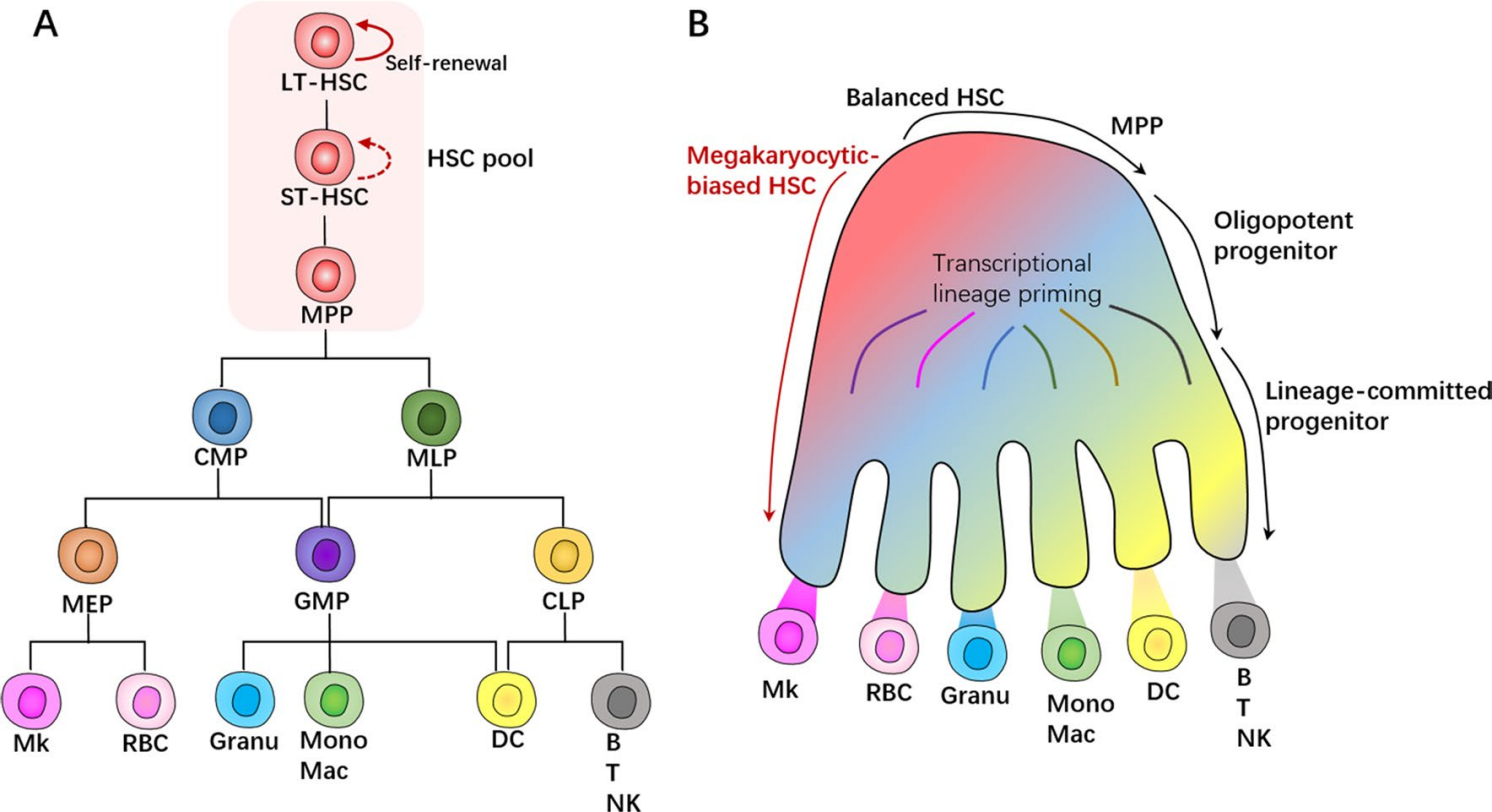
Hierarchical models of hematopoiesis. **A** Representation of one of the classical views of hematopoietic hierarchical tree, showing hematopoietic cells of different potency. The top HSC pool incorporates highly heterogeneous progenitor populations with self-renewal and multipotent differentiation properties, downstream of which the first binary branch point separates the myeloid and lymphoid lineages. Oligopotent cells in the middle and terminally subdivide into different unipotent cells at the bottom by discrete differentiation stages [16, 41]. **B** HSC commitment schematic proposed by single-cell transcriptomic snapshots. The megakaryocytic-biased HSC shows a skewed direct production of Mk while retaining multi-lineage potential. The balanced HSC has equivalent contribution toward the production of all mature blood cells [42, 46]

Present efforts in single-cell molecular profiling are expected to shed light on the fate decision and differentiation landscapes of adult HSCs. Remarkably, the demarcations between stem and progenitor cell populations in the classical view of the hematopoietic hierarchy have been transformed by recent studies focusing on the precise examination of cell-to-cell variation. Single-cell transcriptomic snapshots of cells from the HSPC compartment describe a continuum of hematopoietic differentiation trajectories toward lineage specifications, rather than a stepwise manne [41, 42]. Traditional point of hematopoietic lineage has long been considered as a tree-like hierarchy (Fig. 2A), in which HSCs undergo lineage commitment by generating multipotent progenitors, followed by the subdivision of multipotent progenitor compartment into distinct unipotent subpopulations [43]. This model shows a stepwise restriction of lineage potential at binary branch points. However, due to the functional and molecular heterogeneity of the HSC pool itself, the tree structure is further complicated [41, 44]. A dynamic cell flux model of continuous differentiation landscapes is currently proposed, and that suggests HSCs gradually acquire lineage-committed transcriptomic in a smooth transitional manner, with little or no discrete differentiation stages (Fig. 2B). Wherein multipotent progenitors do not represent discrete cell types but should rather be considered transitory states within the cellular compartment, with two HSCs rarely showing identical patterns [42, 45].

Although multiple systems concerning lineage tracing of individual hematopoietic clone have been reported, they lack simultaneous transcriptome information to describe whether HSC clones with defined fates differ transcriptionally [47–49]. Transcriptional circuits alone do not precisely explain the potential of cells toward distinct fates [50]. The introduction of scRNA-seq into lineages tracing technologies are providing key insight into lineage-associated molecular determinants of HSCs in their native context. A new endogenous RNA barcoding system, developed by Weike Pei and colleagues, integrates lineage information and transcriptome signatures in the same cell, and thus, fate-defined HSCs can be compared on gene expression variability and be positioned onto the transcriptional trajectories [51]. In this dataset, differentiation-inactive HSCs that lack lineage output locate closely to the developmental root compared to multilineage differentiation-active HSCs. Myelo-erythroid-biased HSCs that are closest to multilineage HSCs, reside between myelo-erythroid-restricted and multilineage HSCs. Differentiation-inactive HSCs enriched for LT-HSCs display a large spread in cell-cycle scores, consistent with the long-standing idea that LT-HSC state characterized by the durable self-renewal [9]. Of note, comparation of dormant markers on these HSC subsets breaks through the previous definition of HSC dormancy. Because the evident dormant signatures on differentiation-active HSCs indicate dormant cells also contribute to lineage generation [16], it is limited to define quiescent HSCs with respect to differentiation activity. Toward the same aim, CRISPR-Cas9-based barcoding [31] and expressible lentiviral barcoding approaches [52] also appear advantages on developmental lineage reconstruction, and heterogeneous features of differentiation-active or -inactive HSC clones. These studies further negate the tree-like branching process of hematopoietic differentiation and confirm that cells with similar fate can nonetheless be conferred with different molecular signatures [49].

#### The “lineage-biases” of HSCs

The heterogeneity of cellular compartment is not simply stochastic. Single-cell transplantation has indicated that many cells within the heterogeneous HSC pool actually have different propensities for certain lineage-fate decisions, which are taken earlier than that are expected from the classical hematopoietic tree model [53]. Such “lineage-biases” are quickly consolidated by single-cell transcriptional profiling of human and murine HSCs. It is widely recognized that lineage-biases of HSC change across ontogeny. Early erythroid predominance, and follow-up transition to later lymphoid representation in fetal liver during gestation are considered pivotal for liver hematopoiesis to adapt to the needs of the developing fetus [54]. Tracking of native hematopoiesis describes the predominant native fate of LT-HSCs as megakaryocytic-biased, although their multipotency is unaffected by transplantation [16, 45]. These studies indicate that committing to a megakaryocytic fate may be one of the earliest fate decisions of LT-HSCs. Unlike multipotent progenitors (MPPs), which contribute to the majority of steady-state Ly, Er, and My blood lineage depending on hematopoietic demands, these Mk-lineage LT-HSCs principally generate other blood lineages under extreme conditions, through a ‘direct’ pathway [31, 55, 56].Within the HSC/MPP cloud, HSCs produce in parallel distinct subsets of molecular-biased MPPs, and transcriptional lineage-priming programs toward erythroid/megakaryocytic, myeloid, or lymphoid lineages are already initiated [41, 57]. But HSCs may shift to generate normally rare myeloid-biased MPPs as a transient compartment of myeloid amplification in regenerating conditions with reduced self-renewal activity [43]. Preexisting lineage-specific modules are present at low levels and reinforce along differentiation [56]. In addition, the “lineage-biases” of HSCs are age-dependent. Aged HSCs have high platelet bias priming and reductive lymphoid output, although there is no impact on their potency of engraftment on transplantation [58].

#### Description of cell developmental trajectories

ScRNA-Seq platforms extract the coupling between fates of multiple types of mature cells originating via more than one trajectory in hematopoiesis, as well as putative regulators of fate decision and drivers or inhibitors of differentiation pathways. Through the global transcription landscape of c-Kit^+^ cells, for instance, Tusi et al. reconstructed a dynamic developmental pattern of murine early erythroid trajectory driven by novel erythropoietic regulators (e.g., growth factor receptor *IL-17a*) and uncovered a kind of transcriptional switch that can remodel cell cycle within differentiation. More surprisingly, this reconstructed transcriptional state continuum of HPCs predicted iteratively joining fates of erythroid and basophil/mast cell. Mature monocytes were found to arise from two discrete branches, the dendritic branch and the granulocytic branch [59]. Gene expression dynamics analysis of human bone marrow Lin^−^ progenitors recovered the origin of the basophilic branch among early adult human hematopoiesis. These basophils progenitors were found associated with growth preferences toward Mk (the former) and myeloid (the latter) cell fates. Significantly, scRNA profiling was further utilized to link the early hematopoiesis hierarchical structure of Lin^−^ human population and Kit^+^ mouse BM progenitors. In regard to branching structures, strong similarities have been unveiled among the two organisms. But the different expression signatures of orthologous genes of ribosome biogenesis along the erythroid branch explained the divergence during the human and mouse erythroid differentiation [60]. The cellular hierarchy from a single cell point of view proposes new gating strategies for the isolation of lymphoid/myeloid- and megakaryocyte/erythrocyte-primed progenitor compartments, even HSCs are concluded as existing in a fluid “cloud” (continuum of low-primed undifferentiated hematopoietic stem and progenitor cells) state without a clear separation into single lineages [56, 59, 61].

#### The discovery of new immune cell types

Recent advances on single-cell techniques can also help to uncover rare cell types within a population [62]. Based on the significant expression differences between key erythroid and megakaryocyte genes, the presence of three distinct erythro-megakaryocyte subpopulations, "Pre-MEP", "E-MEP", and a rare population of "Mk-MEP", has been uncovered within classically defined immunophenotypic megakaryocyte-erythroid progenitors (MEPs) in human. These MEP subpopulations can be further discriminated by different gating combinations based on cell surface antigens of CD44, CD41, and CD71 [63]. The dissection of cellular hierarchy of granulocyte-monocyte progenitors (GMPs) identified a previously undescribed intermediate population derived from early committed neutrophil progenitors, which subsequently differentiates into mature neutrophils. Such a neutrophil developmental pathway showed a similarity between mice and human [64]. In-depth analysis of the states of human blood concluded that the intermediate DC (dendritic cell)/monocytes are far more heterogeneous than previously appreciated. New subtypes of human blood dendritic cells and monocytes and the complex relationships between cell types were redefined, including a rare type of DCs that potently activates T cells [65]. Thus, single-cell techniques facilitate more accurate interpretations for heterogeneities and interrelationships of multiple hematopoietic progenitors in immune monitoring in health and disease.

### Understanding the bone marrow microenvironment at single-cell resolution

The bone marrow microenvironment is composed of heterogeneous populations of hematopoietic cells, as well as non-hematopoietic cells that physically associate with HSCs. On the basis of technologies including immunofluorescence confocal or intravital imaging, the localization of HSC has been reported proximal to distinct bone marrow populations in steady state or disease. But the understanding of the relations among adult HSCs and cellular sources of HSC regulators, and their spatial relationships needs to be addressed, largely because of the low frequencies of some rare populations in the BM and the technical challenges associated with their isolation [66]. Recent advances on scRNA-seq enable a precise identification of HSC-niche populations, and these databases that can be interactively browsed will serve as a resource for the future work exploring the bone marrow environment (Table 1).

**Table 1:** Summary of recent findings on dissecting the heterogeneous stromal compartment of bone marrow using scRNA-seq

Publication	Sequencing objects	Key findings	Dataset
Baryawno et al. [67]	Lin^−^Ter119^−^CD71^−^ non-hematopoietic cells	Lepr-MSCs are significant source of Angpt1, Cxcl12, and Kitl New inferred osteoblast differentiation trajectories cause two different osteolineage subsets with distinct hematopoietic support potential A novel fibroblast subset expressing Cxcl12 and Angpt1 Arterial BMECs predominantly express high level of Kitl, Cxcl12 and Vwf compared to sinusoidal and arteriolar BMECs The relative proportions and the expression of hematopoiesis-regulatory factors in these key subsets are impaired by AML	N/A
Wolock et al. [68]	CD45^−^Ter119^−^ non-hematopoietic cells CD31^−^ non-endothelial cells	HSC-supportive CAR cells can be mapped to a single MSC subset within the stroma Newly reconstructed differentiation paths from BM stroma to fat, bone, and cartilage	kleintools.hms.harvard.edu/paper_websites/bone_marrow_stroma
Baccin et al. [69]	Lin^−^CD45^−^CD71^−^stroma cells Lin^−^cKit^+^ hematopoietic cells	Distinct niche residence cells are spatially allocated in the endosteal, sinusoidal, and arteriolar niches Cellular and spatial sources of cytokines to support HSCs Spatial relationships and intercellular signaling interactions of BM resident cell types Novel CAR cell subsets (i.e., Adipo-CAR cells in sinusoidal and Osteo-CAR cells in arteriolar endothelia) Adipo-CAR cells are main source of Cxcl12 and Kitl; Osteo-CAR cells are main source of Csf1 and Il7 Novel Ng2^+^ MSCs being placed at the apex of a differentiation hierarchy for all mesenchymal cell types	http://nicheview.shiny.embl.de
Tikhonova et al. [66]	Col2.3^+^ osteoblasts LepR^+^ perivascular cells VEcad^+^ vascular cells	The majority of niche cells are not actively cycling within quiescent BM microenvironment Adipocytic-primed LepR^+^ cells are preferential source of pro-hematopoietic factors 5-fluorouracil treatment induces cell proliferation across the niche subsets, and impacts adipo-lineage and osteo-lineage differentiation Vascular endothelium is the main sources of Notch ligands Dll4, which prevents the normal myeloid potential of hematopoietic progenitors The expression of vascular endothelial-specific Dll1 and Dll4 is downregulated under acute stress conditions	http://aifantislab.com/niche
Zhong et al. [70]	Endosteal Td^+^ bone marrow cells	Age-dependent changes on the composite of mesenchymal populations and their bi-lineage differentiation routes in young, adult and aging mice A cluster of newly identified large population of adipogenic lineage precursors that regulate marrow vasculature and bone formation 3D networks constructed by Adipoq-labeled stromal cells and pericytes in bone marrow	N/A
Tikhonova et al. [71]	–	An integrated overview of the bone marrow niche derived from five discussed scRNA-seq datasets combined	https://singlecell.broadinstitute.org/single_cell/study/SCP1248

AML, acute myeloid leukemia; BM, bone marrow; BMEC, bone marrow endothelial cell; CAR, CXCL12-abundant reticular; Csf1, colony stimulating factor 1; Cxcl12, C-X-C motif chemokine ligand 2; Dll1, delta like canonical Notch ligand 1; Dll4, delta like canonical Notch ligand 4; Il7, interleukin 7; Kitl: kit ligand; MSC, mesenchymal stem cell; 3D, three-dimensionality; Vwf: Von Willebrand factor; N/A, not applicable

#### Mapping bone marrow niche heterogeneity

The single-cell landscape of the marrow stromal cell populations characterized the expression of important mediators of hematopoiesis (e.g., Cxcl12, Kitl) [68, 72]. Differentiation hierarchies for maturing stromal cells revealed the strongest ‘‘source’’ cell state of the MSCs, as well as some transcription factors of distinct lineages, including the osteocyte, chondrocyte, and adipocyte lineages. Within Lepr^+^ MSPC fraction expressing adipo-gene, a pool of early mesenchymal progenitors (EMPs) with high activity of fibroblastic colony-forming units has been identified [66, 70]. Zhong’ s study showed how these stromal populations, especially the EMPs inside endosteal bone marrow, change over time. Although this cluster of EMPs is responsible for bone formation by mesenchymal lineage cells at all different developmental stages, it drastically shrinks and drifted toward more adipogenic status during age [70]. This study explains the weakening of the marrow activity with increasing age.

**Fig. 3:**
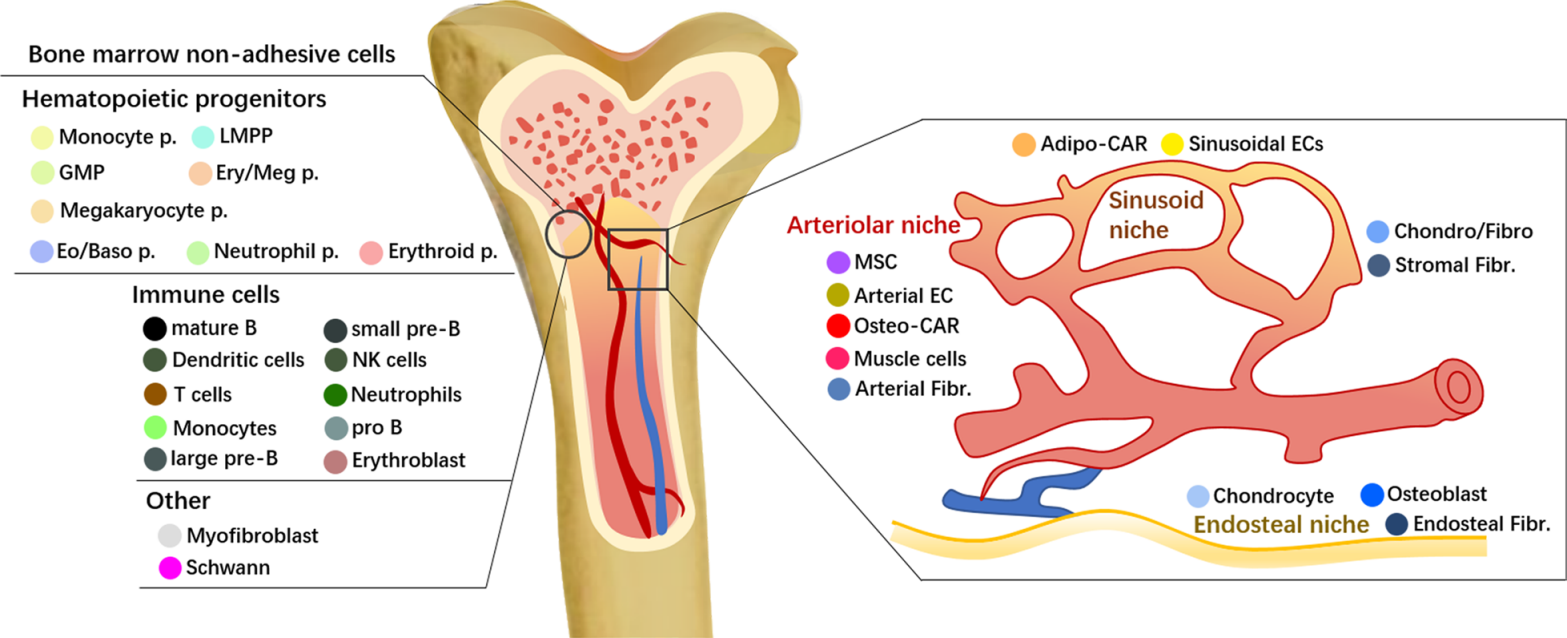
Overview of bone marrow niche cellular composition. ScRNA-seq can be combined with spatial transcriptomics (such as LCM-seq [74]) to reveal the distinct hematopoietic subpopulations as well as visualize spatial allocation of marrow resident cells, revealing the preferential localization of these BM cell types in endosteal niches, sinusoidal niches and arteriolar niches

The integration of single-cell and spatial transcriptomics allows quantitative systematic analyses of HSC localization and spatial organization of the distinct niches (Fig. 3), and such combinations could resolve existing confusion [73, 74]. In total BM, a differentiation continuum spanning megakaryocyte-erythrocyte and lympho-myeloid branches were identified among the major hematopoietic progenitors and immune cell types. Meanwhile, novel subpopulations have been identified in compartments, which have always been regarded as homogenous [8]. For example, scRNA-seq allowed the identification and developmental trajectories of rare non-hematopoietic cells that were previously unrecognized, including two CAR cell subpopulations differentially expressing adipocyte and osteo-lineage genes (e.g., Adipo- and Osteo-CAR cells). In this dataset, spatial relationships and intercellular signaling interactions of BM cell types across niches have been predicted using a newly developed RNA-Magnet algorithm that can measure the specific combinatorial signaling input from different local niches. Different BM resident cell types are successfully allocated to endosteal, sinusoidal, arteriolar, and non-vascular niches. Interestingly, the two newly defined Adipo- and Osteo- CAR cells differ significantly across distinct peri-vascular micro-niches, and serve as not only primary sources of signals interacting to progenitor populations, but the main bone marrow producers of cytokines required for HSCs maintenance and key immunological processes, thereby establishing special key sites for HSCs in perivascular niches [73, 75]. Similar bioinformatic methods based on the expression of cell adhesion molecules have been recently developed. They contribute greatly to decoding ligand-target links across interacting cells of BM [73, 76] or other tissues [77]. Going beyond cell–cell communication mediated by ligand-receptor pairs, existing tools have successfully been designed to (1) settle heteromeric complexes between two cell types (e.g., CellPhoneDB [78]), (2) curate classification of ligand-receptor pairs into related signaling [79], (3) infer downstream intracellular signaling (NicheNet [80]), (4) assess cellular communities that are communicating the most specifically (NATMI [81], and (5) map the spatial organization of BM microenvironment (RNA-Magnet mentioned above [73]). They will explain how the fates of various niche populations are intertwined to regulate HSC survival and behaviors, and ultimately build three-dimensional cellular hierarchies and crosstalk within BM microenvironment.

Notably, simultaneous detection of RNAs and proteins admonished that some niche populations have high expression of several cytokines at the transcriptome level, but their protein expression seems significantly lower [82]. Thus, the profiling of the bone marrow environment at the protein level should be further considered when interpreting the inherent interactions of niche cells on the basis of transcriptome data. Nevertheless, perspectives from scRNA-seq are able to provide rich information on the temporospatial-specific bone marrow niches and inspire the functional experiments to further explore HSCs niche architecture.

#### Stress-induced changes in bone marrow niche

Within steady bone marrow stroma, the majority of niche cells are non-cycling cells [66]. Mesenchymal, pericyte, fibroblast and endothelial subpopulations express distinct hematopoietic regulatory genes. Remodeling of the bone marrow stroma in leukemia provides a significant distorted stromal compartment model where parenchymal cross-communicate with stromal elements to facilitate the emergence of cancer [67].

Previous studies have tried to describe how hematopoiesis support cells orchestrate the hematopoietic regeneration in BM niche after high doses of cytotoxic agents and sought for ways circumventing this toxicity [83, 84]. Tikhonova et al. further revealed a previously unappreciated level of molecular reprogramming of the BM microenvironment after the chemotherapy treatment of leukemia [66]. Stromal cellularity and expression of pro-hematopoietic factors show high sensitivity to stress, resulting in a premature myeloid transcriptional program. The newly discovered adipocytic skewing of the perivascular cell under chemotherapy-treated conditions contributes to exploring the cellular mechanisms which govern the increase in the frequency of the BM adipocytes [85]. A protein-based sing-cell expression mapping further confirmed the stress-induced dynamic changes in stromal cell populations and cytokine production. In addition, proteomic analysis revealed that irradiation insult led to the loss of LeptinR^+^ and Nestin^+^ subsets, whereas CD73 expressing stromal cells persist post-irradiation, being considerable mediators of HSPC engraftment and hematopoietic reconstitution [86].

### Toward clinical application of single-cell analysis in hematological disease

The hematopoietic homeostasis within the organism is primally determined by the ability of HSCs to self-renew, differentiate, or remain quiescent, thereby producing a balanced output of all of the required mature blood cells to compensate for system perturbations such as injury and infection. But hematological malignancies hijack hematopoiesis, resulting in increased proliferation and/or a differentiation block [87, 88]. Cancer driving events and clonal heterogeneity are undoubtedly controlled by both genetic composition and gene expression. ScRNA-seq technologies pave the way to unravel intra-tumoral heterogeneity, but these efforts are still nascent because this technology lack the sensitivity required to reliably detect somatic mutations. These questions could be addressed by some recent emerging complementary single-cell methods. The integrative analysis of somatic mutations with transcriptional states has been a popular method to clearly probe genotype–phenotype relationships and illustrate how malignancy-driving mutations initiate and sustain disease.

#### Resolving intra-tumoral heterogeneity (ITH)

Cancer stem cells (CSCs), such as leukemia stem cells (LSCs), are the cells responsible for giving rise to a spectrum of leukemia subpopulations to initiate and propagate the disease [89]. Researchers have highlighted the application of scRNA-seq to study the heterogeneity of LSCs of various leukemia subtypes so that a more precise understanding of clonal evolution can be achieved [90]. Bulk RNA sequencing technologies fail to define the extent to which mutations drive heterogeneity in leukemia populations. To resolve this current limitation, scRNA-seq is required to dissect the composition of cell types populating each sample and define cellular locations of mutation. The work of Lavalle et al. is an excellent and representative example of these studies, presenting a comprehensive single-cell profiling of primary acute myeloid leukemia (AML) caused by NPM1 mutations. A subpopulation of cells that have acquired NPM1 mutations was demonstrated, and the emergence of acquired mutations could be found in cells with different lineage commitment in different patients [91].

Linking the transcriptional heterogeneity with mutation frequency in different cell types provides genetic and cellular basis that guides the progression from normal cells to these important leukemia subpopulations. Combining of single-cell transcriptional landscape with mutational status offers an unprecedented opportunity to uncover the impact of somatic mutations on cellular phenotype. A pioneering approach by Wang et al. sensitively detected DNA- and RNA-level information of chronic lymphocytic leukemia (CLL) samples. Interestingly, this approach unexpectedly found novel CLL drivers (mutated LCP1 and WNK1) that that would be missed by the analysis of bulk samples. This single-cell analysis suggested the phenotypic convergence between distinct subclones despite different genetic identities [92].

Virus infection, as a well-known causative agent for murine and avian myeloid leukemias, affects multistep process during tumor evolution; however, the infectious etiology has not been definitively established for human AML. Transcriptomic landscapes at a single-cell level increase the likelihood of any minute viral RNA transcripts in tumor progenitors. Various virus has been identified on bone marrow cell samples from AML patients. Compared with non-CD34 cells, more viral RNA transcripts prefer to harbor in CD34 cells, where these virus alter oncogenes or tumor-suppressor genes and cause mutations, and these mutations in the progenitor cells could then be passed down to the cells it would differentiate to [93]. The application of scRNA-seq is expected to further discern crucial information about virus tumorigenicity, assisting the better diagnostics or patients in different stages of infection [94].

#### Exploring resistant mechanisms in malignant hematopoiesis

Recent studies have revealed the presence of self-renewing LSCs with distinct self-renewal gene expression profiling in NRAS^G12V^-driven AML mouse model and human AML samples. Self-renewal and rapid proliferation are indeed separate functions in these LSCs as they are in normal HSCs, being the primary cause of frequent relapse in patients following antiproliferative therapies, as well the main obstacle to cure in AML. The identification of “LSC-specific” molecular signature highlights the importance of targeting self-renewal, in addition to proliferation, to eliminate the true LSCs and prevent relapse in AML [95]. BMP pathway related mechanisms by which TKI-persisting LSCs arise and evolve upon treatment were subsequently illustrated. High levels of BMPR1B receptor in LSCs promoted the formation of a permanent pool of LSCs, which had quiescence and stem cell signatures. Blocking BMPR1B/Jak2 signal in this LSCs could be additional targeting strategy to reduce palindromic cases [96].

#### Advantages of single-cell RNA-based targeted analysis in malignant hematopoiesis

The existence of rare therapy-resistant CSCs subset impedes a complete disease elimination and patients remain at risk of disease relapse. These residual CSCs are typically outnumbered by their normal tissue counterparts, from which they cannot easily be reliably separated [97]. Therefore, it becomes important to characterize these residual CSCs and their novel properties that evolved as a result of the therapeutic selection process. In Giustacchini’s study, a new protocol that integrates fluorescence-activated cell sorting (FACS), high-sensitivity single-cell mutation detection and scRNA-seq were integrated to develop a new protocol for the separation of BCR-ABL^+^ SCs from nonclonal HSC compartment within individual patients with chronic myeloid leukemia (CML) [98]. This technology offered an added resolution on previously unrecognized heterogeneity of CML-SC, and the distinct clustering of normal HSCs, BCR-ABL^**+**^ and BCR-ABL^**−**^ SCs, including the identification of a rare subset of BCR-ABL^+^ SCs with the selective persistence following TKI treatment. Parallel mutation analysis highlighted the importance of insights into deregulated pathways of mutant and non-mutant cells, and thereby predicted cellular mechanisms conferring therapy resistance [98].

Myelofibrosis is the most life-threatening symptom of the myeloproliferative neoplasms (MPNs), a group of heterogeneous disorders that result from constitutive activation of JAK2 signaling through somatic mutations affecting JAK2, MPL, or CALR. Unlike the rarity of megakaryocytes in healthy bone marrow, mutant-clone HSPCs in MF dramatically increase megakaryocyte numbers, with the release of excess pro-fibrotic cytokines and growth factors, and even progress to AML [99]. Different combinations of multiple mutations that collectively cause a malignant phenotype contribute to substantial genetic and bone marrow microenvironment complexity in MF. Unlike Tyrosine Kinase Inhibitors (TKI) in CML, survival of patients with MF is not substantially improved by currently available JAK inhibition therapies since the possibility of appearance of unrelated clones over time [100]. Therefore, a major focus of drug developments is identifying novel targets beyond JAK inhibitors [101]. In this regard, a novel single-cell ‘‘multi-omics’’ method, including scRNA-seq, targeted single-cell mutational analysis with simultaneous scRNA-seq (TARGET-seq) and single-cell proteomics, has advanced ultimate reconstruction of tumor phylogenetic trees. Abnormal megakaryopoiesis identified from early multipotent stem cells in MF and unique myelofibrosis megakaryocyte progenitors (MkPs) expressing aberrant molecular signatures has been revealed. Megakaryocyte-induced fibrosis is possibly due to both normal expansion of megakaryocytes and the generation of an aberrant population. Unprecedented insight into effective treatment algorithm for MF has been provided. G6B expression on MkPs suggested the early validation of G6B as a potential immunotherapy target. The identification of novel MF drivers that not previously implicated as regulators of megakaryocyte differentiation suggested additional targets for inhibiting myelofibrosis clone while preserving erythropoiesis [102]. This work serves as proof of observation that would not have been possible without analysis of gDNA and cDNA alongside scRNA-seq.

## Conclusions and outlook

The past few years have witnessed the great effectiveness of scRNA-seq analyses on tracing the origin of HSCs, profiling the hematopoietic hierarchy, drawing the hematopoietic microenvironment of bone marrow and providing a practical basis for the diagnosis and treatment of hematologic malignancies. But with the rapid development of this technology, substantial obstacles have been encountered in this field.

First, owing to cumbersome operation and current limited efficiency of reverse transcription, future studies are likely to make further use of the current efforts to increase the efficiency of RNA capture and cell throughput with lower detection cost, so that the use scope of sc-RNA seq can be broaden to more basic research [11, 103]. Second, cell states from dissociated tissue may be affected by mechanical and enzymatic dissociation, or fluorescence-activated cell sorting in many gene expression studies. Since the effects of these artificial procedure contaminate the cellular transcriptomes and induce a “dissociation-affected subpopulation” [104], the results of previous relevant studies where dissociation procedures have been used may need to be reinterpreted; future optimization on cell dissociation should thus be developed to ameliorate the changes and to promise a temperate isolation of intact, unperturbed single cells. Finally, the most cell maps solely show information about polyadenylated mRNAs; there are remaining challenges of ongoing technical improvements to non-polyadenylated transcript classes, such as regulatory non-coding RNA (e.g., miRNAs, lncRNAs, circRNAs, snoRNAs, or piRNAs) [105, 106]. Together with multi-omics, such as single-cell COOL-seq [107], scNMT-seq [108], and scTrio-seq [109], and scM&T-seq [110], these multi-dimensional inspection strategies can provide comprehensive information that helps to paint a more multifaceted landscape of each cell in developmental hematopoiesis. Accordingly, improvement of computational approaches is hoped to connect, integrate and interpretate single cell datasets across studies [71, 111]. Undeniably, scRNA-seq technologies are able to continue a trend which we believe will have critical impact on the biological basis of HSCs.

## Data Availability

Data sharing is not applicable to this article as no datasets were generated during the current study.

## Abbreviations

AGM: Aorto-gonad-mesonephros
AML: Acute myeloid leukemia
BM: Bone marrow
BMEC: Bone marrow endothelial cell
CAR: CXCL12-abundant reticular
CLL: Chronic lymphocytic leukemia
CLPs: Common myeloid progenitors
CML: Chronic myeloid leukemia
CMPs: Common lymphoid progenitors
CSCs: Cancer stem cells
DC: Dendritic cell
ECM: Extracellular matrix
EHT: Endothelial‐to‐hematopoietic transition
EMPs: Early mesenchymal progenitors
FACS: Fluorescence-activated cell sorting
FL: Fetal liver
GMP: Granulocyte-monocyte progenitor
HE: Hemogenic endothelial
HECs: Hemogenic endothelial cells
HPCs: Hematopoietic progenitor cells
hPSCs: Human pluripotent stem cells
HSCs: Hematopoietic stem cells
HSPCs: Hematopoietic stem and progenitor cells
IAHC: Intra-aortic hematopoietic cluster
LncRNA: Long non-coding RNA
LSCs: Leukemia stem cells
LT-HSC: Long-term HSC
MEPs: Megakaryocyte-erythroid progenitors
MkPs: Megakaryocyte progenitors
MPN: Myeloproliferative neoplasm
MPPs: Multipotent progenitors
MSCs: Mesenchymal stem cells
ScRNA-seq: Single-cell RNA sequencing
TFs: Transcription factors
TKI: Tyrosine kinase inhibitor
YS: Yolk sac
